# Mechanistic insights into MARK4 inhibition by galantamine toward therapeutic targeting of Alzheimer’s disease

**DOI:** 10.3389/fphar.2023.1276179

**Published:** 2023-09-19

**Authors:** Mohd Adnan, Debarati DasGupta, Saleha Anwar, Anas Shamsi, Arif Jamal Siddiqui, Mejdi Snoussi, Fevzi Bardakci, Mitesh Patel, Md Imtaiyaz Hassan

**Affiliations:** ^1^ Department of Biology, College of Science, University of Ha’il, Ha’il, Saudi Arabia; ^2^ College of Pharmacy, University of Michigan, Ann Arbor, MI, United States; ^3^ Centre for Interdisciplinary Research in Basic Sciences, New Delhi, India; ^4^ Centre of Medical and Bio-Allied Health Sciences Research, Ajman University, Ajman, United Arab Emirates; ^5^ Research and Development Cell, Department of Biotechnology, Parul Institute of Applied Sciences, Parul University, Vadodara, India

**Keywords:** kinase inhibitors, acetylcholinesterase inhibitors, Alzheimer’s disease, isothermal titration calorimetry, molecular dynamics simulation

## Abstract

**Introduction:** Hyperphosphorylation of tau is an important event in Alzheimer’s disease (AD) pathogenesis, leading to the generation of “neurofibrillary tangles,” a histopathological hallmark associated with the onset of AD and related tauopathies. Microtubule-affinity regulating kinase 4 (MARK4) is an evolutionarily conserved Ser-Thr (S/T) kinase that phosphorylates tau and microtubule-associated proteins, thus playing a critical role in AD pathology. The uncontrolled neuronal migration is attributed to overexpressed MARK4, leading to disruption in microtubule dynamics. Inhibiting MARK4 is an attractive strategy in AD therapeutics.

**Methods:** Molecular docking was performed to see the interactions between MARK4 and galantamine (GLT). Furthermore, 250 ns molecular dynamic studies were performed to investigate the stability and conformational dynamics of the MARK4–GLT complex. We performed fluorescence binding and isothermal titration calorimetry studies to measure the binding affinity between GLT and MARK4. Finally, an enzyme inhibition assay was performed to measure the MARK4 activity in the presence and absence of GLT.

**Results:** We showed that GLT, an acetylcholinesterase inhibitor, binds to the active site cavity of MARK4 with an appreciable binding affinity. Molecular dynamic simulation for 250 ns demonstrated the stability and conformational dynamics of the MARK4–GLT complex. Fluorescence binding and isothermal titration calorimetry studies suggested a strong binding affinity. We further show that GLT inhibits the kinase activity of MARK4 significantly (IC_50_ = 5.87 µM).

**Conclusion:** These results suggest that GLT is a potential inhibitor of MARK4 and could be a promising therapeutic target for AD. GLT’s inhibition of MARK4 provides newer insights into the mechanism of GLT’s action, which is already used to improve cognition in AD patients.

## Introduction

The stability of microtubules is controlled by microtubule-associated proteins (MAPs) and tau protein (Ramkumar et al., 2018). MARK kinases can phosphorylate serine motifs in the microtubule-binding domain (MBT) of tau, and this phosphorylation is critical in the dynamics of microtubules. Microtubule-associated regulating kinase 4 (MARK4) is an evolutionarily conserved Ser/Thr kinase (Doerflinger et al., 2003; Goldstein and Macara, 2007) that phosphorylates classical MAPs, namely, tau and others (Illenberger et al., 1996; Trinczek et al., 2004). MARK kinases phosphorylate MAPs, and this phosphorylation results in conformational changes, altering the association of MAPs with microtubules to regulate the microtubule dynamics. Hyperphosphorylation of tau is a key pathological feature in Alzheimer’s disease (AD), and studies have established the critical role of MARK4 in this event (Annadurai et al., 2017). Tau is phosphorylated at Ser262 by MARK4, leading to its detachment from the microtubules, making itself available for phosphorylation by other kinases (Drewes et al., 1997; Mandelkow et al., 2004). According to the literature, MARK4 is associated with defects in synapses and dendritic spines (Yu et al., 2012). These studies establish the significance of MARK4 in neurodegenerative diseases (NDs).

Protein kinases govern various signaling pathways, and thus, their aberrant expression directly contributes to the pathology of cancers, NDs, and other disorders (Shchemelinin et al., 2006; Ahamad et al., 2023). The overexpression of MARK4 is associated with many oncogenic signaling pathways, such as NF-κB, mTOR, Wnt, and Akt (Li and Guan, 2013; Liu et al., 2016; Tian et al., 2019), signifying the importance of this kinase. The kinase is also associated with the growth and metastasis of cancer cells by upregulating the Hippo signaling pathway (Heidary Arash et al., 2017). MARK4 also regulates miR515-5p, implicated in metastasis and cancer cell migration. A recent study reported that MARK4 could improve myocardial function after myocardial infarction by preventing heart failure by microtubule detyrosination (Yu et al., 2021). Another study established the importance of MARK4 in regulating biological processes, namely, glucose and energy homeostasis (Sun et al., 2012). Thus, the aforementioned reports indicate that MARK4 is an attractive druggable target for cancers, neurodegeneration, and other disorders (Anwar et al., 2022c; Voura et al., 2023).

A report signified the implications of acetylcholinesterase inhibitors (AChEIs), rivastigmine tartrate, and donepezil in AD therapeutics targeting MARK4 (Shamsi et al., 2020a). Galantamine (GLT) was initially launched in the United States and Europe as an anti-AD drug sold under the brand name Reminyl^®^ by Janssen Pharmaceuticals (Mucke, 2015). GLT is derived mainly from plants of the *Amaryllidaceae* family and can be synthesized chemically. GLT belongs to the alkaloid of the phytochemicals and is majorly present in the bulbs and aerial parts of plants such as *Galanthus* spp. and *Leucojum* spp. (Heinrich and Teoh, 2004).

There are various hypotheses regarding the disease’s onset and progression, including a decrease in acetylcholine (ACh) levels. Inhibitors of ACh catabolic enzymes, acetylcholinesterase (AChE), including GLT, have contributed to increased ACh levels in the brain (Marucci et al., 2021). Thus, this study investigated the MARK4 inhibitory potential of GLT as the MARK4 is associated with pathological phosphorylations of the tau protein, which further contributes to AD progression (Noble et al., 2013). This moderate AChE inhibitor was isolated from *Galanthus woronowi* (Berkov et al., 2007) and is an allosteric inhibitor of nicotine receptors (Coyle and Kershaw, 2001). In AD patients, improvement in cognition and function can be achieved through GLT therapy; GLT therapy results in the stabilization of cognitive performance (Sharma, 2020). Thus, we aimed to see whether GLT can be exploited as a MARK4 inhibitor to control AD and tauopathies.

This work explored the binding affinity of GLT to MARK4 by employing various computational and experimental methods (Parveen et al., 2018; Mohammad et al., 2019a; Naqvi et al., 2020). Fluorescence studies revealed the MARK4–GLT complex to be stable with appreciable binding affinity. Moreover, isothermal titration calorimetry (ITC) revealed the binding energetics and thermodynamics of the MARK4–GLT system. Additionally, molecular docking and molecular dynamics (MD) simulations were carried out to decipher the conformational dynamics of the MARK4–GLT system and provide atomistic details of the interaction of GLT with MARK4.

## Materials and methods

### Materials

GLT was bought from Merck (Germany). Luria broth (M575) was procured from HiMedia Laboratories, and Ni-NTA resin (Cat. No./ID: 30,210) was obtained from Qiagen. All chemicals were of the highest analytical quality. Isopropyl β-D-1-thiogalactopyranoside (IPTG) and Tris were obtained from Himedia. Double distilled water was used for buffer preparations, and all the buffers were filtered before usage.

### Molecular docking

We conducted molecular docking analysis to identify the residues that play a critical role in MARK4–GLT interaction. A high-resolution structure (2.4Å) with no mutations of MARK4 (PDB ID 5ES1) was used in the study. We created a receptor grid for blind docking as center coordinates (X = −38.71 Y = −14.92 Z = −2.162) with dimensions of 60 × 72 × 83 Å. We positioned the grid box on the centroid of the protein, which was enough to investigate the MARK4 structure for the ligand. We followed all other steps mentioned in the earlier published literature (Anwar et al., 2022a). We carried out docking using our in-house tool InstaDock (Shafie et al., 2021), and the top nine obtained poses were thoroughly investigated. The best pose was in terms of interaction with critical residues of the protein subjected to MD simulations as described (Khan et al., 2023).

### MD simulations for testing the stability of the docked complex

We optimized the geometry of the fragment using the B3LYP/6-31G(d) method in the Gaussian 16 program (Frisch et al., 2016) to obtain GLT parameters. The electrostatic potential (ESP) charges were computed using Merz-Kollman radii (Singh and Kollman, 1984). All other steps were followed, as mentioned in the earlier published literature (Anwar et al., 2022b). MD simulations employed GPU-accelerated Amber20 (Götz et al., 2012). A time step of 2fs was used in the study, and SHAKE (Ryckaert et al., 1977) was turned on. All other steps were carried out in our earlier communications (Anwar et al., 2022b).

### Molecular mechanics generalized Born surface area analysis

The molecular mechanics generalized Born surface area (MMGBSA) approach harnesses force-field methodologies to estimate molecular systems’ binding free energy (Δ*G*_bind_), with ligand–receptor complexes being a prime example. This energy is typically represented in kcal/mol. In our study, the MMGBSA module from AmberTools facilitated the determination of the binding free energy for the docked complex. After the MD simulations, we sampled frames from the MD trajectories at 10 ns intervals. The cumulative binding free energy was then derived using the following equation:

$$∆G_{\text{bind}}=G_{\text{complex}}-\left(G_{\text{protein}}+G_{\text{ligand}}\right),$$

where ∆*G*_bind_ = binding free energy, *G*_complex_ = free energy of the protein–ligand complex, *G*_protein_ = free energy of the protein, and *G*_ligand_ = free energy of the ligand.

### Expression and purification of MARK4

MARK4 was expressed and purified following our published protocol (Khan et al., 2017). The purity of protein was checked on SDS-PAGE.

### Fluorescence measurements

After ascertaining the binding of the MARK4–GLT system using computational approaches, the next step was to find the real affinity of the binding of GLT with MARK4. Fluorescence spectra were recorded on a Jasco FP-6200 spectrofluorometer using a 5 mm quartz cuvette. The protein concentration used for the measurement was 4 µM. The experimental parameters used were as follows. We excited the protein at 280 nm, and emission was recorded in the 300–400 nm range with the excitation and emission slit width set at 10 mm and response set to medium. Each reported scan was an average of three scans. MARK4 was titrated with varying GLT concentrations (0–0.9 µM), and the obtained data were analyzed using modified Stern–Volmer (MSV) equations as per the earlier published literature (Anwar et al., 2020a; Khan et al., 2021).

### Isothermal titration calorimetry

We carried out the ITC experiment to decipher the binding energetics of the MARK4–GLT system and find the associated thermodynamic parameters of the MARK4–GLT complex as per earlier studies (Shamsi et al., 2020a; Alhumaydhi et al., 2021). Initially, all the solutions were degassed for 30 min to ensure no bubbles in the samples that could hinder the reaction or create a problem in obtaining the actual binding parameters. There are two cells: the sample cell and the reference cell. The protein of interest, MARK4 (20 μM), was filled in the sample cell, while the corresponding buffer was filled in the reference cell. The ligand, GLT (200 μM), was loaded in the rotating syringe and the program was set for 25 injections into the sample cell with spacing between two injections set at 180 s and the time duration of all injections was kept at 20s. The first injection (5 µL) was false, followed by successive 20 µL with a reference power of 16 μcal s^-1^. We used four site binding models to analyze the raw data, and the final figure was plotted using MicroCal Origin 8.0 software.

### Kinase inhibition assay

The kinase assay is a “malachite green reagent-based” microtiter plate assay. We performed a kinase assay to ascertain the kinase inhibitory potential of GLT as per earlier published studies (Anwar et al., 2021). Initially, we incubated the protein of interest (MARK4 with 6 µM concentration) with increasing GLT concentrations in a 96-well plate for 1 h at 25°C. After that, we prepared and added ATP to the reaction mixture with 10 mM MgCl_2_. After addition, we leave the mixture undisturbed for 30 min at 25°C. Then, we added the “BIOMOL reagent (200 μL)” to stop the reaction, and the mixture was left at room temperature for 20 min for color development. We then transferred 100 μL of the final reaction mixture to 96-well plates read at 620 nm on a Multiskan FC ELISA reader.

## Results and discussion

### Molecular docking

**FIGURE 1:**
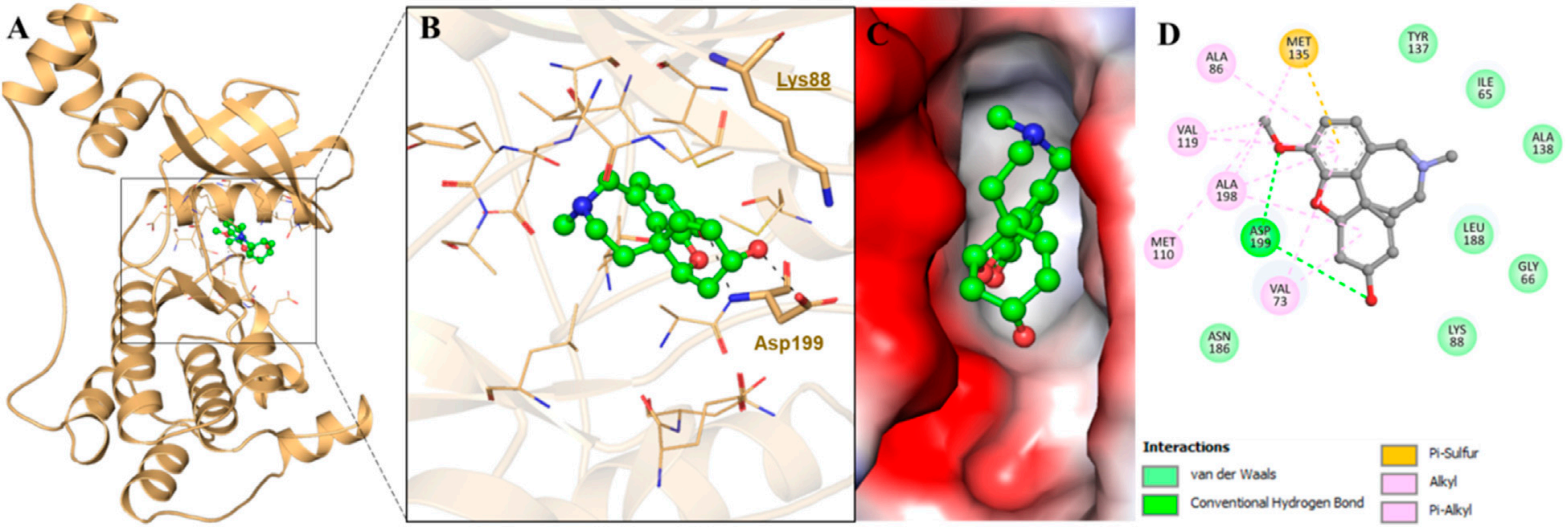
Showing interaction of MARK4 with GLT. **(A)** Cartoon representation, **(B)** interacting residues, **(C)** potential surface cavity, and **(D)** 2D structural representation of MARK4 residues interacting with GLT.

We further performed a detailed molecular docking analysis to demonstrate the binding sites and relevant molecular interactions playing a key role in MARK4–GLT interaction. The docking results showed that GLT exhibited an appreciable binding affinity of −8.6 kcal/mol. It was revealed that GLT resides in the ATP-binding pocket of the kinase (Figure 1A). It interacts directly with MARK, exploiting its ATP site residues, which include Lys88 and further Asp199, forming two hydrogen bonds (Figure 1B). It lies in the deep cavity of the MARK4 and is forming several interactions with the key residues of the binding pocket (Figure 1C). The detailed analysis of the ligand–protein interaction suggested that GLT interacts with the ATP-binding pocket of MARK4 with the involvement of several functionally active residues. It was also observed that Asp199, which has roles as a crucial residue for MARK4 functionality, offers dual interactions as conventional hydrogen bonding to the ligand GLT (Figure 1D). Concurrently, several other interactions were also formed, such as Met135 of the protein kinase, which was found to be involved in a single pi–sulfur bond. Some residues involve shared alkyl bonds with many van der Waals interactions with GLT (Figure 1D).

### MD simulations

Protein structure fluctuations inside a protein can be correlated with its genetic behaviors (Sharma et al., 2019). Structural alterations in the enzyme, whether minor or major, can significantly affect the activity (Khan et al., 2019). Inhibiting the enzymes by small molecules is essential in several pathways, such as infectious pathways and/or pathways leading to cancer. Therefore, conformational modifications and structural variations are subjected to assessments with the effect of the inhibitory activity of these compounds (Khan et al., 2018; Naqvi et al., 2018). Small-molecule ligands (SMLs), when bound to their respective proteins targeted, can exhibit substantial fluctuations in the confirmation. MD simulations are of utmost importance to trace these phenomena at the atomic level. For evaluation of the stability, several parameters such as root-mean-square fluctuation (RMSF), root-mean-square deviation (RMSD), and H-bonding such as intermolecular and intramolecular H-bonding in the kinase–ligand complexes were subjected to evaluation from the simulated trajectories.

**FIGURE 2:**
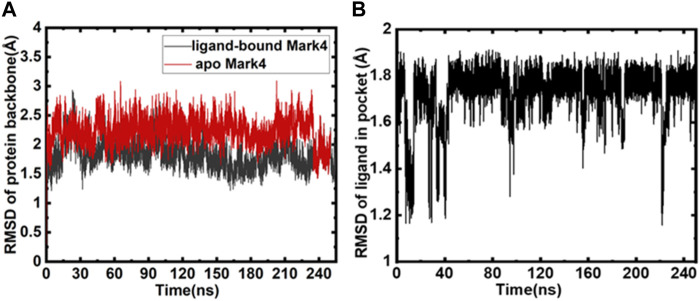
**(A)** Backbone RMSD of protein (red) and GLT-bound MARK4 (black); **(B)** RMSD of GLT as a function of time.

We calculated all the MD trajectories for GLT-bound MARK4 and free MARK4. The RMSD of the free protein and protein bound to GLT was equated (Figure 2A), and RMSD plots show a substantially stable protein backbone during the simulation. The average RMSD was <2.5 Å away from the initially solvated complex. GLT’s RMSD was also assessed for its movement analysis during the 250 ns run (Figure 2B). The results emphasize that GLT binding to the protein marginally stabilizes the complex, as apparent from the red RMSD trajectories of the ligand-bound MARK4 compared to the apo form.

### Analysis of intra/intermolecular hydrogen bonding

**FIGURE 3:**
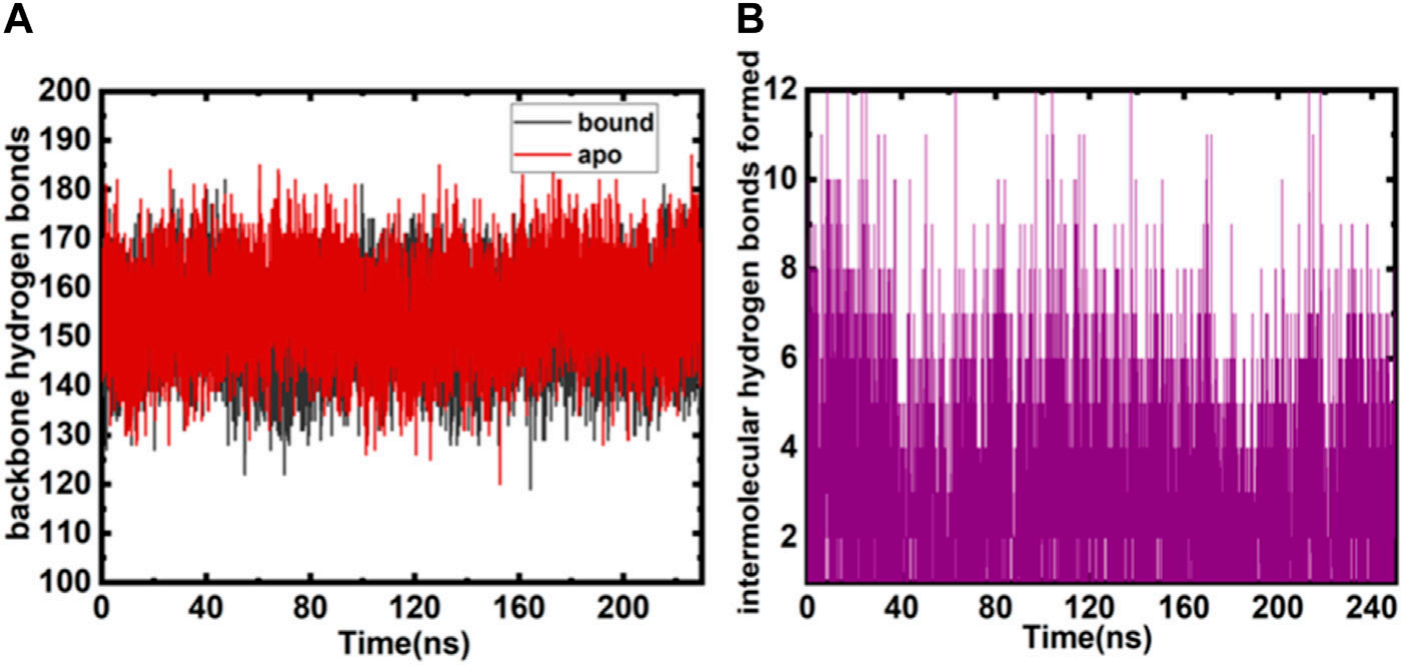
**(A)** Intramolecular backbone hydrogen bonds (both free and bound MARK4) plotted as a function of the number of snapshots. **(B)** Intermolecular hydrogen bonds were monitored between bound and MARK4 protein and GLT.

Various parameters evaluate the overall protein structure and its conformational stability; one such parameter is the role of intramolecular and intermolecular hydrogen bonds (Mohammad et al., 2019b; Voura et al., 2019). The evaluations exhibit an overall insight into the enzyme–ligand associations. The intramolecular hydrogen bonds (MARK4 backbone) formed during GLT binding and free protein do not show remarkable deviations and appear consistent (Figure 3A). The intermolecular hydrogen bonds between the protein–ligand were also calculated during the simulation, and six to seven main hydrogen bonding interactions appear consistently throughout the 250 ns run. The hydrogen bonds between GLT and MARK4 are plotted in Figure 3B. The key hydrogen bonding residues interacting with GLT are Lys-88, Glu-90, Asp-147, Glu-133, Leu-136, and Asp199.

### Free energy analyses

Computational chemists are interested in estimating the binding affinity of the ligand with the target protein globally. Calculating the binding free energy is important for any biophysical process. Enumerating binding affinities for small molecules has emerged as a major hurdle. Various methodologies have been subjected to evolution in the past few decades to advanced techniques of computational free energy calculations. The perturbation of free energy is a benchmark in calculating free energy; however, this method is exhaustive and demands huge monetary support. For this study, we employed the MMGBSA technique and linear interaction energy (LIE) methods (Aqvist et al., 2002; Almlöf et al., 2007). In the last 3 decades, MMGBSA/PBSA methods have evolved, and this evolution has resulted in minimizing the computational time required to perform detailed calculations, albeit with few approximations. Using adjustable parameters α and β, the LIE method accurately estimates the net electrostatics and van der Waals interaction of a ligand binding to the protein.

**FIGURE 4:**
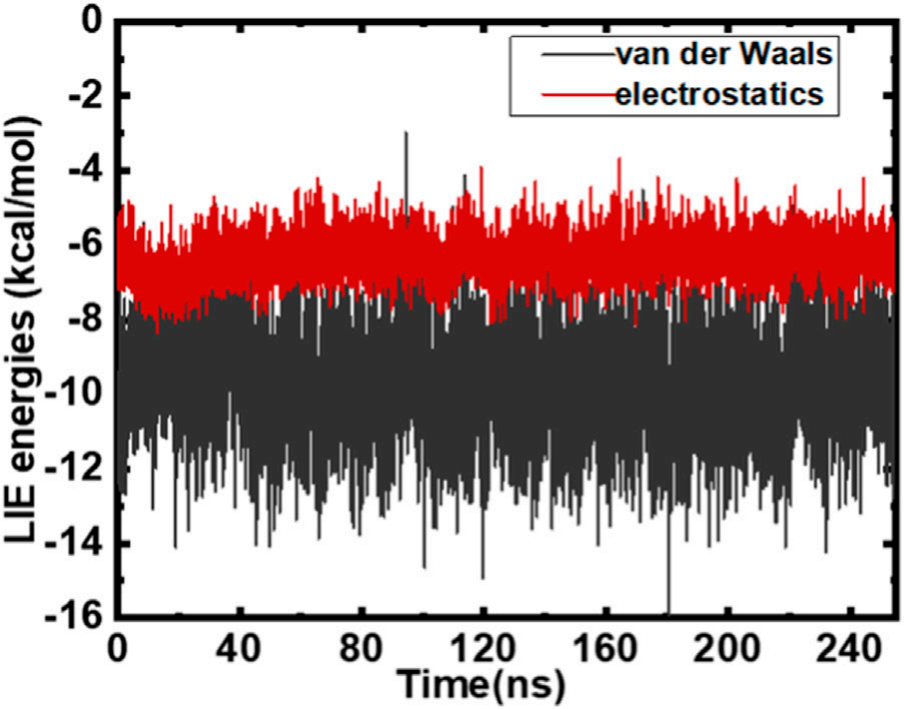
LIE estimates plotted as a function of time post-scaling energies (kcal/mol).

We estimated electrostatic and van der Waals interaction energies. The MMGBSA technique and LIE methods (Aqvist et al., 2002; Almlöf et al., 2007) were employed to study the MARK4–GLT complex. LIE is fairly precise in calculating the overall interactions (electrostatic and van der Waals) of a ligand associated with a protein. It is scaled using α and β as parameters. Interaction energies from both interactions were estimated for the MARK–GLT complex. The overall changes in the components and interactions between GLT and MARK4 are plotted and depicted in Figure 4. The net Δ*G*_binding_ was calculated as −18.8 kcal/mol. AmberTools (Roe and Cheatham, 2013) processed the trajectory for estimating MMGBSA and PBSA energies. LIE underestimated the binding affinity values when equated with MMGBSA. The difference was −31.2 kcal/mol estimated with MMGBSA, and the LIE estimate was −18.8 kcal/mol. Both estimates were found to be nearby and within the limit of error.

### Fluorescence binding assay

**FIGURE 5:**
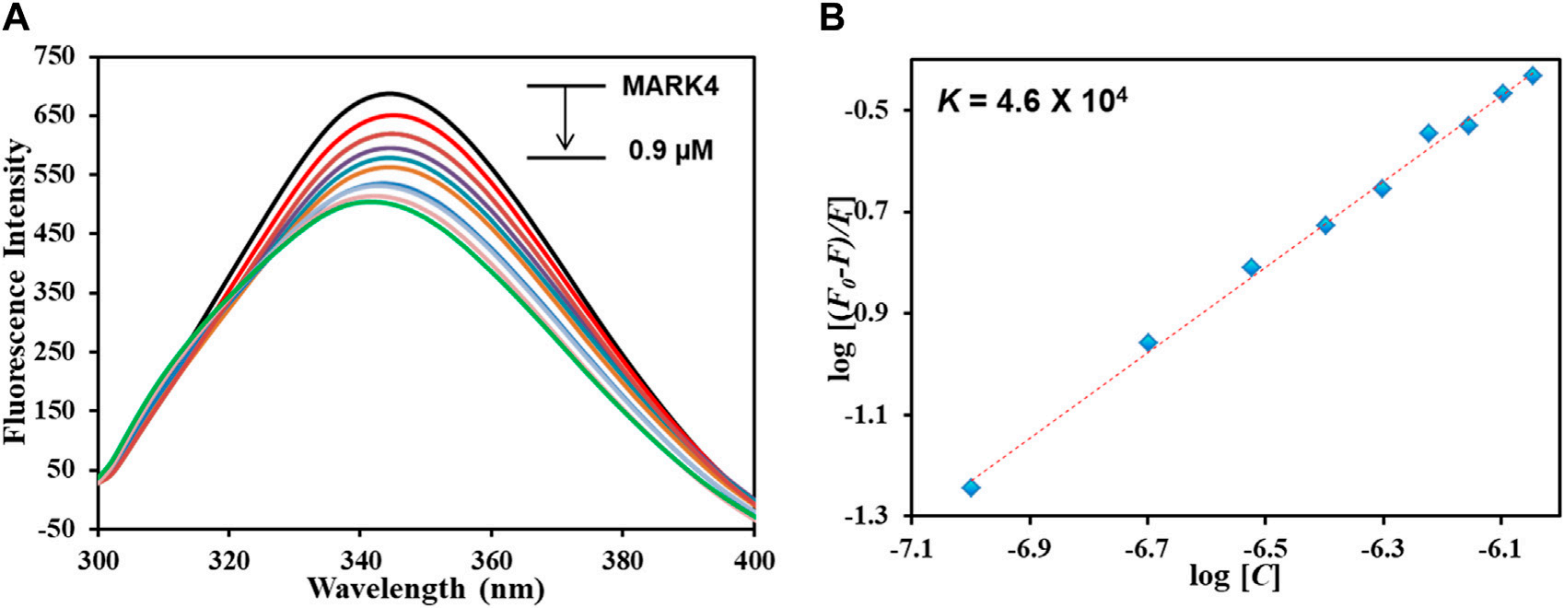
**(A)** Fluorescence emission spectra of MARK4 alone and in the presence of GLT concentrations (0–0.9 µM). **(B)** MSV plot of the MARK4–GLT system.

An important tool for analyzing protein–ligand complexes is fluorescence spectroscopy (Klajnert et al., 2003; Soares et al., 2007) due to its importance in revealing the actual binding of a ligand with the protein and computing various binding parameters. When a ligand interacts with the protein, it results in the quenching of fluorescence that is retorted to obtain multiple binding parameters. The phenomenon of fluorescence quenching arises from numerous reasons: molecular shuffling, interactions between molecules, transfer of charges amongst excited states, and complex formation between the protein and ligand in the ground state. Figure 5A illustrates the fluorescence emission spectra of MARK4 (ligand-free) and MARK4 with increasing GLT concentrations (0–0.9 µM); it is evident that MARK4 fluorescence intensity decreased with an increase in concentrations of GLT, showing that GLT suppresses the fluorescence of the protein. The emission spectra show the formation of a stable MARK4–GLT complex. The obtained data were equated with the MSV equation to assess various binding parameters of the complex.

Figure 5B depicts the modified SV plot; the binding constant denoted with *K* was derived from the intercept of this plot, and the number of binding sites (*n*) was estimated from the slope. The binding constant (*K*) was estimated to be 4.6 × 10^4^ M^-1^, showing that GLT associates with MARK4 with a decent affinity, generating a stable complex and validating *in silico* results. Comparable magnitudes of the binding constants have been noticed for other protein–ligand complexes, representing a strong binding (Anwar et al., 2020a). Reports of inhibitors of MARK4 also show binding constants of a similar magnitude. Thus, it can be interpreted that GLT associates with MARK4 with an appreciable binding affinity, forming a stable protein–ligand complex.

### Isothermal titration calorimetry

**FIGURE 6:**
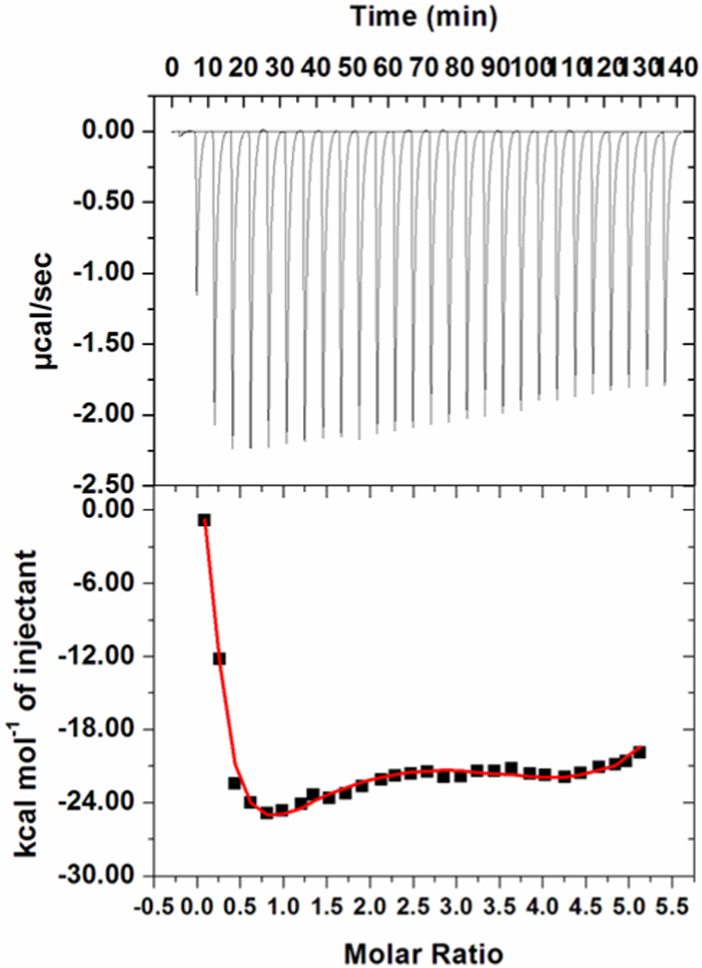
ITC profile of the MARK4–GLT system. The top panel shows raw data obtained upon sequentially titrating the GLT solution into MARK4. The bottom panel shows the binding isotherm obtained upon plotting the integrated heat results of the calorimetric titration after correction of heat of dilution against the molar ratio.

ITC is a sophisticated biophysical technique employed for thermodynamic analysis of systems binding or interacting with each other in a free solution (Shamsi et al., 2020a; Shamsi et al., 2020c). Heat is used as a signal to determine several parameters used in thermodynamic life changes in free energy, enthalpy, entropy, and binding affinity. The interactions between the protein and ligand are estimated by assessing the variations in enthalpy that play a major role in the drug discovery process (Aneja et al., 2019; Voura et al., 2019). An isotherm generated for the MARK4–GLT system is shown in Figure 6. GLT associates with the protein spontaneously, as apparent from the isotherm generated from sequential injections of GLT into the protein. The upper section of the figure shows negative heat pulses, which suggest an exothermic binding. The lower section depicts the liberated amount of heat corresponding to each ligand injection. The thermodynamic parameters obtained are shown in Table 1. These parameters were obtained by fitting the ITC isotherm in a four-binding site model.

**TABLE 1:** Thermodynamic parameters obtained from best-fitted ITC isotherm of the MARK4–GLT system.

*K*_a_ (association constant) M^-1^	∆*H* (enthalpy change) cal/mol	∆*S* (cal/mol/deg)
*K*_a1_ = 1.03 × 10^5^ ± 1.1 × 10^4^	∆*H*_1_ = 7,238 ± 2.51 × 10^3^	∆*S*_*1*_ *=* 47.2
*K*_a2_ = 7.88 × 10^4^ ± 7.2 × 10^3^	∆*H*_2_ = −2.03 × 10^5^ ± 1.91 × 10^4^	∆*S*_*2*_ *=* −659
*K*_a3_ *=* 8.60 × 10^4^ ± 8.1 × 10^3^	∆*H*_3_ = 2.77 × 10^5^ ± 3.13 × 10^4^	∆*S*_*3*_ *=* 952
*K*_a4_ *=* 6.78 × 10^4^ ± 7.5 × 10^3^	∆*H*_4_ = −2.82 × 10^5^ ± 1.71 × 10^4^	∆*S*_*4*_ *= -*925

### Kinase inhibition assay

**FIGURE 7:**
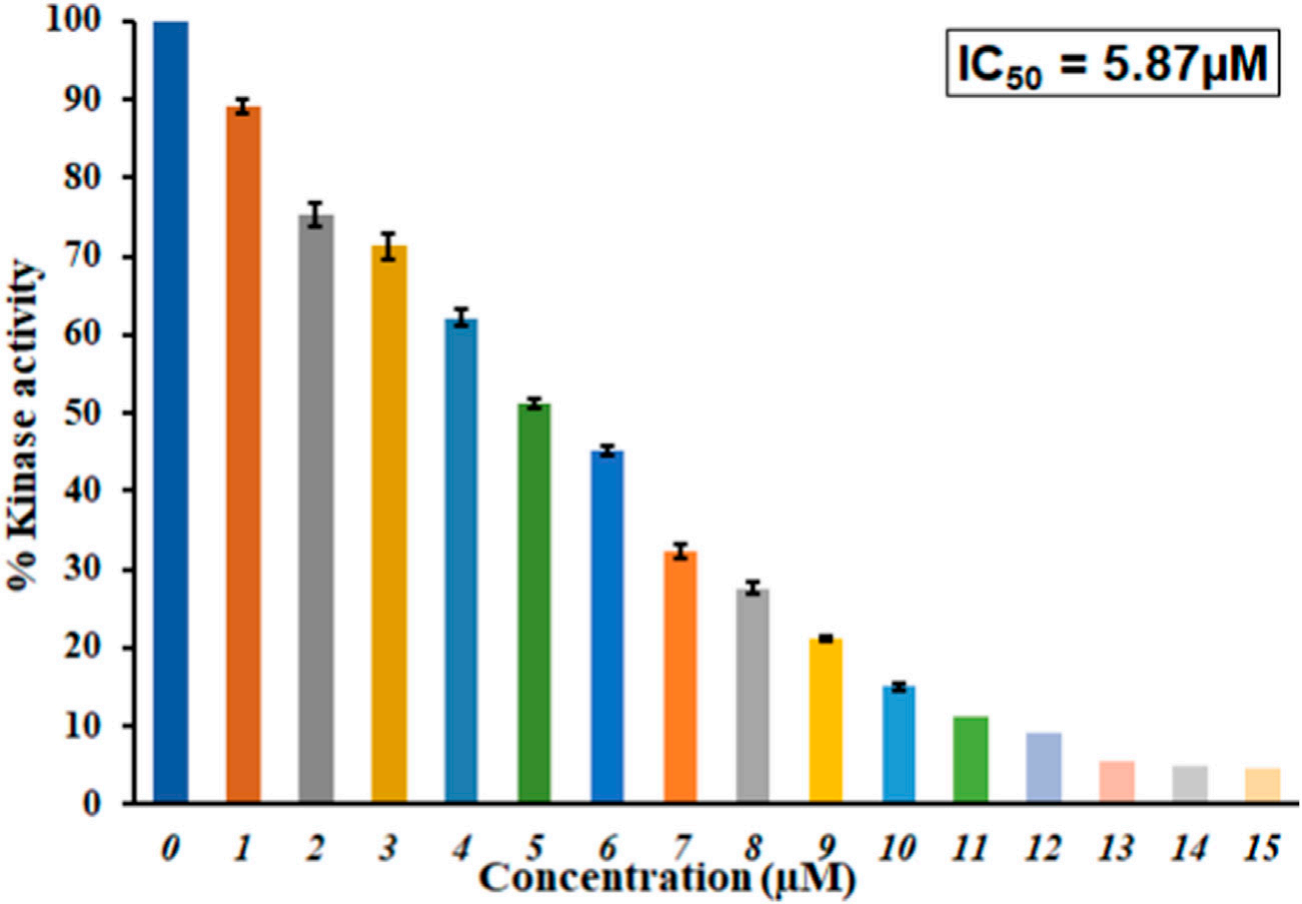
Inhibition of ATPase activity of MARK4 by GLT. Percent inhibition in the ATPase activity of MARK4 as a function of GLT concentration (0–15 µM).

An enzyme inhibition assay is considered to assess a ligand’s potential against a protein’s activity and functionality. With a malachite green-based ATPase assay, we investigated GLT’s inhibitory potential toward the MARK4 kinase activity. The kinase inhibition activity of the ligand GLT is also shown in Figure 7. The concentration of GLT was taken from 0 to 15 µM. It was observed that MARK4 activity shows a progressive decrease with an increase in GLT concentration.

The inhibitory concentration for 50% inhibition of the kinase, IC_50_, was estimated to be approximately 5.87 µM. This reveals that GLT inhibits 50% of the MARK4 kinase activity at 5.87 µM. Comparing IC50 values with earlier inhibitors suggests that GLT is a potent MARK4 inhibitor with an IC_50_ of similar magnitudes reported for other inhibitors in recent studies highlighting the importance of GLT (Shamsi et al., 2020a; Anwar et al., 2020b). Several recently published studies reported MARK4 inhibitors (Anwar et al., 2021; Shamsi et al., 2022), and studies targeting the identification of kinase inhibitors are in great demand due to key pathways governed by the kinases. Thus, it is evident that GLT binds to MARK4, forms a stable complex, and inhibits the kinase activity of MARK4; hence, it can be implicated in managing MARK4-directed diseases.

These observations corroborate our molecular docking, MD simulation, fluorescence, and ITC measurements, confirming that GLT binds to MARK4 with a good affinity forming a stable complex and inhibiting its kinase activity, i.e., GLT may be a potent inhibitor of MARK4. We assume that GLT inhibits the hyperphosphorylation of tau and the following formation of NFTs via inhibition of the activity of MARK4 (Gotz et al., 2001; Giovinazzo et al., 2021; Moore et al., 2023).

## Conclusion

Due to the immense importance of MARK4 in cancers, AD, and other disorders, it is gaining significant attention as a druggable target. Many studies target to inhibit kinases, as these regulate critical steps of various important cellular signaling pathways. The overexpression of MARK4 is directly linked to the hyperphosphorylation of tau, which is a critical event in AD pathology contributing to AD development. One of the phosphorylation sites on tau is Ser262 where MARK4 preferentially phosphorylates. Phosphorylation at Ser262 is associated with pathological tau phosphorylation and causes tauopathies, including AD.

Various synthetic and natural compounds (Anwar et al., 2020c; Anwar et al., 2021; Anwar et al., 2022d; Voura et al., 2022; Adnan et al., 2023) have been used in the past, showing their potential role as MARK4 inhibitors. Anti-AD agents such as donepezil and rivastigmine have also been studied for their mechanistic roles on AD *via* inhibiting MARK4 activity (Shamsi et al., 2020b). This study established GLT, a drug in use for AD therapy, as a MARK4 inhibitor. This work sheds light on the mechanism of action of GLT and how it works in AD treatment, i.e., GLT inhibits MARK4 activity, which in turn, prevents tau hyperphosphorylation. It is known that GLT, AChEI, improves cognition in AD patients; inhibition of MARK4 by the same drug suggests a common targeting during AD therapy. New molecules with improved affinity and selectivity against MARK4 can be designed for AD treatment.

## Data Availability

The original contributions presented in the study are included in the article/Supplementary Material; further inquiries can be directed to the corresponding author.

## Abbreviations

AD, Alzheimer’s disease; GLT, galantamine; ITC, isothermal titration calorimetry; MARK4, microtubule-associated regulating kinase 4; and MD, molecular dynamics.
